# Supplementary figures and images for: Regional medical inter-institutional cooperation in medical provider network constructed using patient claims data from Japan
Source: PLoS One. 2022 Aug 24;17(8):e0266211. doi: 10.1371/journal.pone.0266211 (PMC9401144; doi:10.1371/journal.pone.0266211)

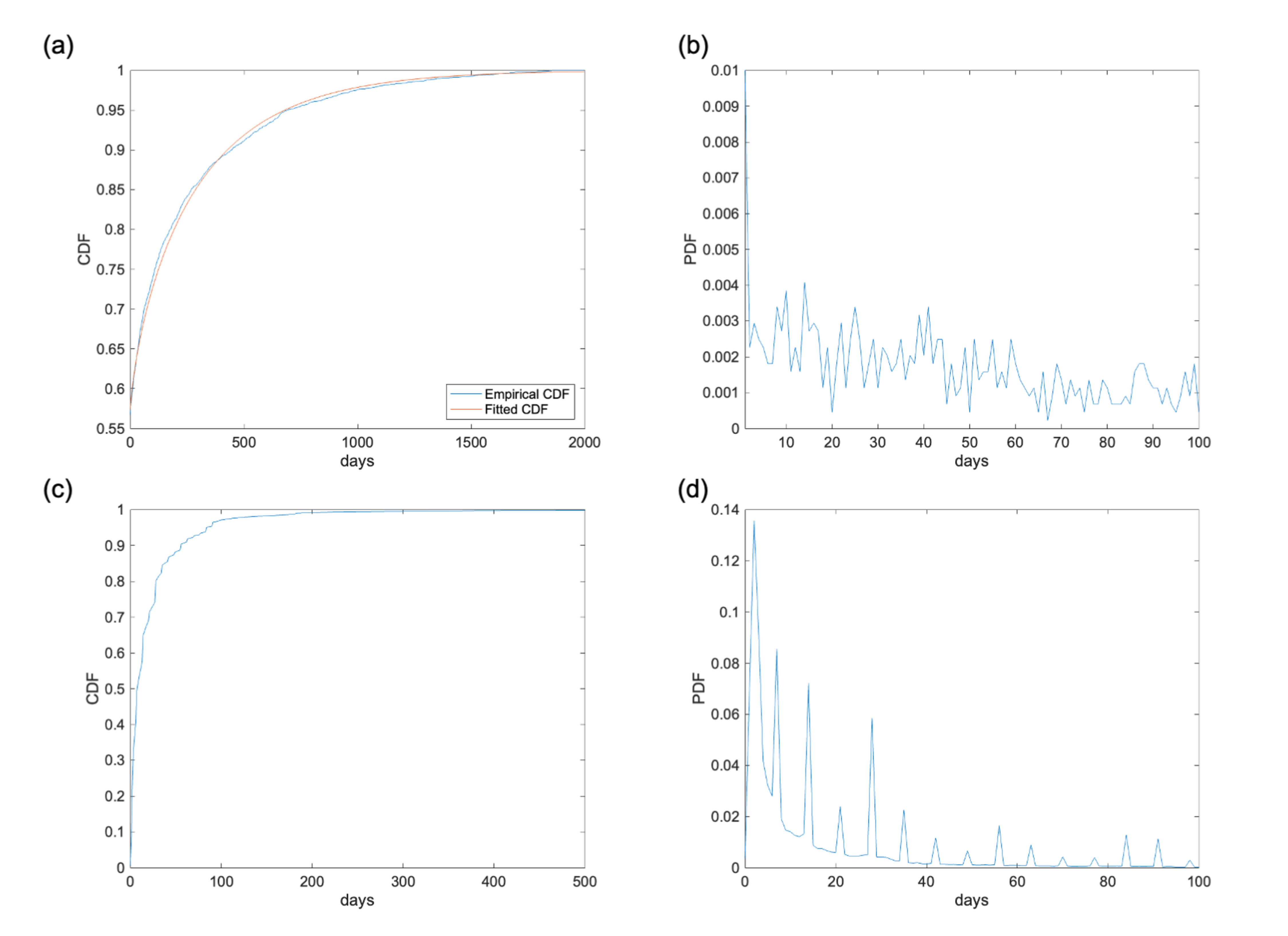

Supplement: S1 Fig — (a) Cumulative distribution function (CDF) of inpatient intervals. (b) Probability distribution function (PDF) of inpatient intervals. (c)Cumulative distribution function (CDF) of outpatient intervals. (d)Probability distribution function (PDF) of inpatient interval. The distribution of inpatient intervals followed an exponential distribution (μ = 126), except for the zero-day interval. The outpatient interval has a cyclic variation with a peak every 7 days. The inpatient interval distribution included approximately 60% of the inpatient interval at τ1 = 10 days. The outpatient interval distribution included approximately 80% of the outpatient interval at τ2 = 35 days. (TIF) [file pone.0266211.s001.tif]

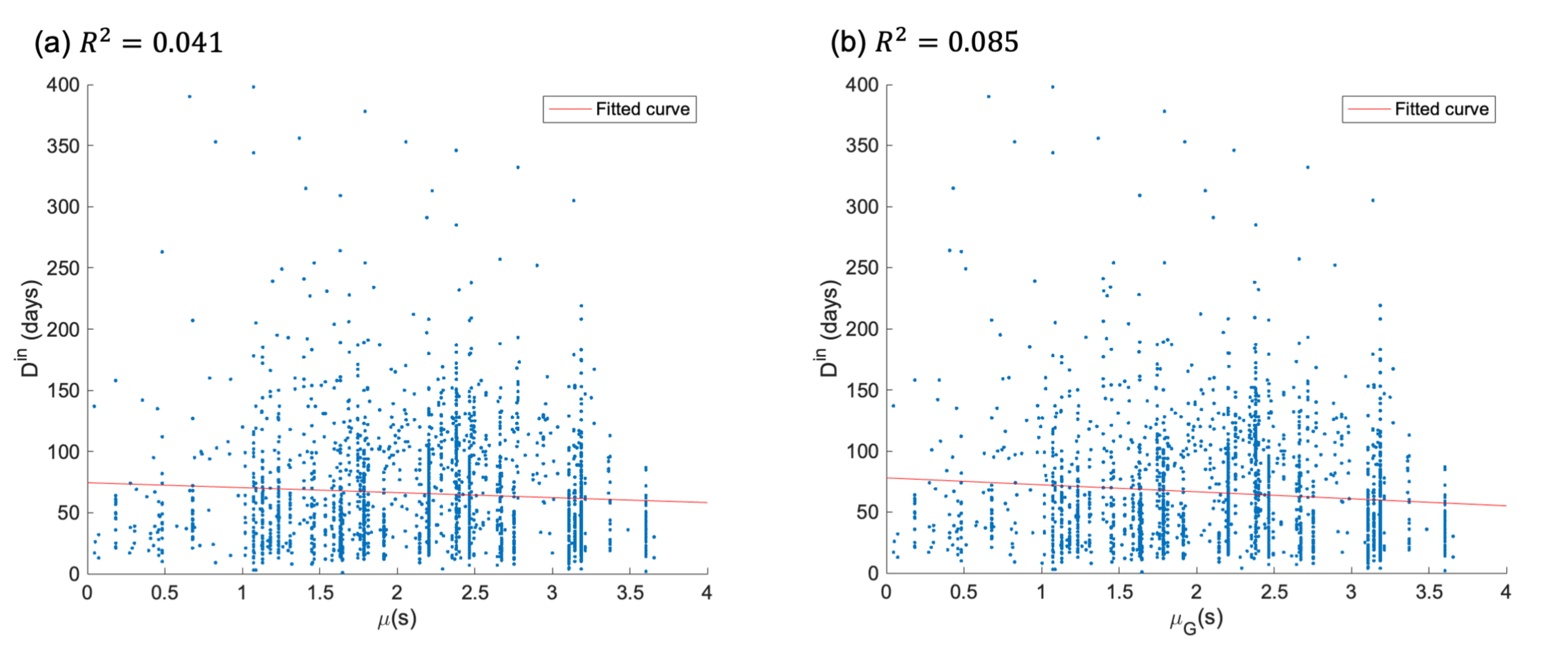

Supplement: S2 Fig — (a) shows mean μ(s). (b) shows the geometric mean μG(s). The geometric mean has a better explanatory ability when both R2 values are compared. (TIF) [file pone.0266211.s002.tif]

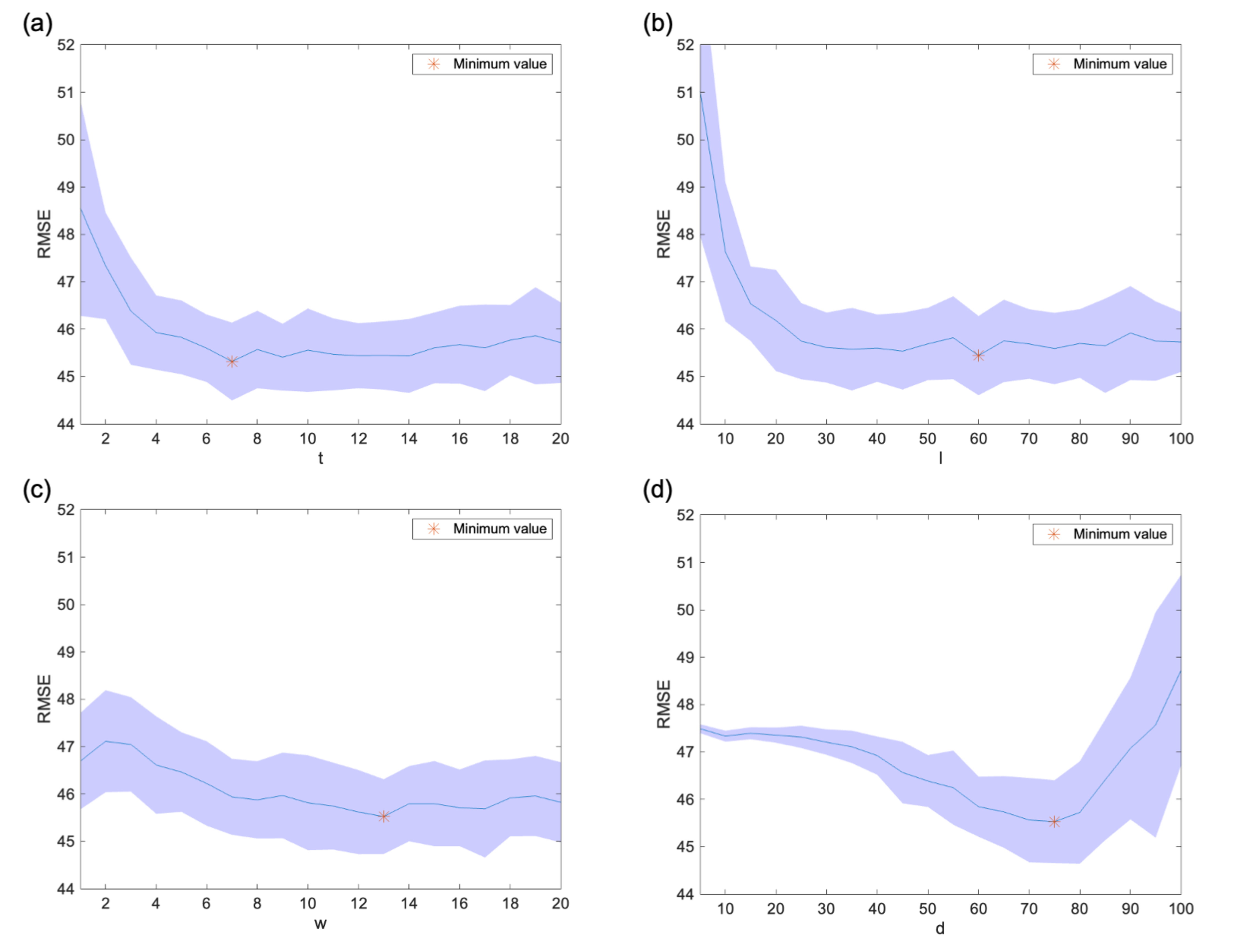

Supplement: S3 Fig — (a) number of walkers per node t; (b) Length of random walk l; (c) Window size w; and (d) Dimension d. We set the default parameter values as (t, l, w, d, p, q) = (5, 25, 10, 70, 1, 1), and extracted feature representations by adjusting t, l, w, and d by one parameter each. We performed a regression analysis using the extracted v. We calculated the root mean squared error (RMSE) using D^in obtained from regression analysis. We performed 20 iterations with five-fold cross-validation and used the average value of the RMSE as the evaluation value of the regression performance. (TIF) [file pone.0266211.s003.tif]

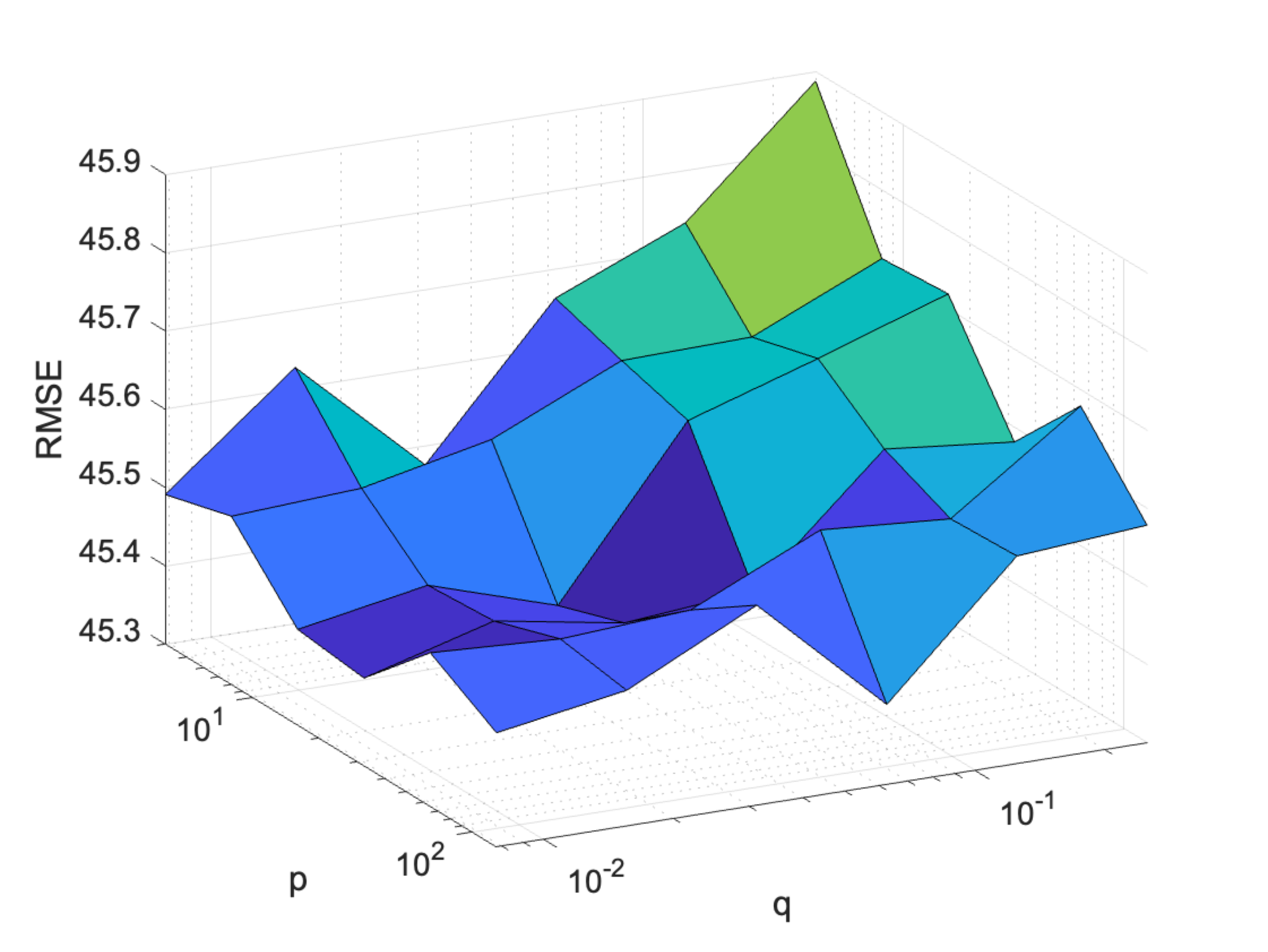

Supplement: S4 Fig — We evaluated the regression performance with changes in parameters p and q using the RMSE. The figure shows that the RMSE approaches the plane when p increases, and q decreases. This means that there are a large number of local minima, which makes it difficult to find a unique optimum value. (TIF) [file pone.0266211.s004.tif]
